# Isotopic Fractionation of Sulfur in Carbonyl Sulfide by Carbonyl Sulfide Hydrolase of *Thiobacillus thioparus* THI115

**DOI:** 10.1264/jsme2.ME17130

**Published:** 2017-12-02

**Authors:** Takahiro Ogawa, Shohei Hattori, Kazuki Kamezaki, Hiromi Kato, Naohiro Yoshida, Yoko Katayama

**Affiliations:** 1 Graduate School of Agriculture, Tokyo University of Agriculture and Technology 3–5–8 Saiwai-cho, Fuchu, Tokyo 183–8509 Japan; 2 Department of Chemical Science and Engineering, School of Materials and Chemical Technology, Tokyo Institute of Technology 4259 Nagatsuta-cho, Midori-ku, Yokohama, Kanagawa 226–8502 Japan; 3 Graduate School of Life Sciences, Tohoku University 2–1–1 Katahira, Aoba-Ku, Sendai, Miyagi 980–8577 Japan; 4 Earth-Life Science Institute, Tokyo Institute of Technology 2–12–1–IE–1 Ookayama, Meguro-ku, Tokyo 152–8550 Japan

**Keywords:** biogeochemical processing of sulfur, sulfur isotopes in COS, GC-IRMS, sink of atmospheric COS, β-class carbonic anhydrase

## Abstract

Carbonyl sulfide (COS) is one of the major sources of stratospheric sulfate aerosols, which affect the global radiation balance and ozone depletion. COS-degrading microorganisms are ubiquitous in soil and important for the global flux of COS. We examined the sulfur isotopic fractionation during the enzymatic degradation of COS by carbonyl sulfide hydrolase (COSase) from *Thiobacillus thioparus* THI115. The isotopic fractionation constant (^34^*ɛ* value) was −2.2±0.2‰. Under experimental conditions performed at parts per million by volume level of COS, the ^34^*ɛ* value for intact cells of *T. thioparus* THI115 was −3.6±0.7‰, suggesting that, based on Rees’ model, the ^34^*ɛ* value mainly depended on COS transport into the cytoplasm. The ^34^*ɛ* value for intact cells of *T. thioparus* THI115 was similar to those for *Mycobacterium* spp. and *Williamsia* sp., which are known to involve the conserved region of nucleotide sequences encoding the clade D of β-class carbonic anhydrase (β-CA) including COSase. On the other hand, the ^34^*ɛ* value was distinct from those for bacteria in the genus *Cupriavidus*. These results provide an insight into biological COS degradation, which is indispensable for estimating the COS global budget based on the isotope because of the significant contribution of COS degradation by microorganisms harboring β-CA family enzymes.

Carbonyl sulfide (COS) is the most abundant sulfur compound in the troposphere at nearly 500 parts per trillion by volume (pptv) (8, 53). Due to its chemical stability, some COS in the troposphere is transported to the stratosphere and is ultimately converted to sulfate by photolysis or a reaction with O or OH radical (7, 9). The resultant sulfate is one of the major sources of the stratospheric sulfate aerosols influencing the Earth’s radiation balance and ozone depletion (9, 75). On the other hand, COS has been proposed as a promising tracer to estimate gross primary production by plants (5, 70, 77). Thus, it is imperative to understand the global budget of COS in order to estimate climate change by stratospheric sulfate aerosols in the future. However, estimates of COS emission and uptake values remain unclear (42).

Major sinks of atmospheric COS are vegetation and soil, and soil microorganisms, such as bacteria and fungi, are considered to play an important role in COS degradation in soil because many isolates belonging to diverse taxonomic groups exhibit strong COS-degrading abilities (48, 56). β-class carbonic anhydrase (β-CA, EC 4.2.1.1) (57), COSase (55), and carbon disulfide (CS_2_) hydrolase (EC 4.4.1.27) (71, 72) exhibit high COS-degrading activities and belong to the β-CA family of enzymes, which have similar amino acid sequences and X-ray crystal structures. Among these enzymes, COSase purified from *Thiobacillus thioparus* THI115 is the only bacterial enzyme with characterized catalytic properties and an elucidated X-ray crystal structure (55). *T. thioparus* is a chemolithoautotrophic sulfur-oxidizing bacterium that widely inhabits soil and freshwater (37). *T. thioparus* THI115 was isolated from activated sludge used for the wastewater treatment of a coke-oven factory, and can grow using thiocyanate, which is an ingredient in the wastewater, as a sole energy source (34). During thiocyanate degradation, COS is produced as a reaction product of thiocyanate hydrolase (EC 3.5.5.8), and the resultant COS is then hydrolyzed to hydrogen sulfide and carbon dioxide by COSase, with COS ultimately being oxidized to sulfate (34, 35, 38, 55). Thus, COSase is an important enzyme in energy production by *T. thioparus* THI115.

An isotope analysis represents a promising tool for tracing the global dynamics of atmospheric trace gases that influence the Earth’s climate (4, 27). In order to apply an isotope analysis and interpret changes in isotopic compositions on an observational scale, the assessment of isotopic fractionation in chemical/biological processes is an essential step. In this context, studies examining isotopic fractionation in each process have involved incubation experiments with isolates or natural environmental samples. However, large variabilities have been reported in isotopic fractionation factors, even for the same reactions, due to differences in the experimental conditions used (*e.g.* [32, 83] for sulfur oxidation). Thus, the examination of isotopic fractionation via isolated enzymes is important for comparing and discussing the factors controlling it. To the best of our knowledge, the study of isotopic fractionation by isolated enzymes involved in biogeochemical reactions has been limited to enzymes such as RubisCO for photosynthesis (59), glycolate oxidase for photorespiration (18), nitrate reductase (33) and nitric oxide reductase (81) for denitrification, hydroxylamine oxidoreductase (81) for nitrification, nitrogenase (76) for nitrogen fixation, and glutamate dehydrogenase (80) and glutamine synthetase (82) for ammonium assimilation. In the case of biogeochemical sulfur cycles, isotopic fractionation has only been examined using dissimilatory sulfite reductase (DsrAB), one of the important enzymes in microbial sulfate reduction (43). Although previous studies reported isotopic fractionation using isolates and/or natural samples (6, 13–16, 19, 26 [cited from 6 and 83], 28–32, 41 [cited from 83], 50, 51 [cited from 6 and 83], 54, 79, 83), an enzyme level analysis has not yet been conducted on isotopic fractionation for sulfur oxidation.

Online gas chromatograph-isotope-ratio mass spectrometry (GC-IRMS) for COS sulfur isotopic measurement was recently developed and has the ability to measure sulfur isotopic compositions at nanomole COS levels (22). By using this method, isotopic fractionation constants for COS degradation at 4,000 parts per million by volume (ppmv) COS were assessed using chemoorganotrophic COS-degrading soil bacteria (36) isolated by Kato *et al.* (29). Four strains of *Mycobacterium* spp., *Williamsia* sp., and two strains of *Cupriavidus* spp. showed a preference for the degradation of CO^32^S over CO^34^S, with isotopic fractionation constant (^34^*ɛ*) values of −3.99 to −3.56‰, −3.74‰, and −2.38 to −2.09‰, respectively. Although the number of isolates used in these experiments was limited, these findings suggest that the differences observed in ^34^*ɛ* values among the isolated bacteria are highly dependent on the genus. Although experiments at very low concentrations, such as atmospheric COS, cannot be conducted for technical reasons, elucidating the mechanisms controlling isotopic fractionation will contribute to estimations of isotopic fractionation by microbial COS degradation in natural environments. Therefore, the isotopic fractionation constants of COSase, which is a unique enzyme for COS degradation identified in the chemolithoautotrophic sulfur-oxidizing bacterium of *T. thioparus* THI115, as well as those of intact cells of this bacterium were assessed in the present study.

We examined the sulfur isotopic fractionation of COS by COSase in consideration of the importance of β-CA family enzymes to the global COS budget. Furthermore, sulfur isotopic fractionation in COS degradation by intact cells of *T. thioparus* THI115 was investigated in order to clarify the details of isotopic fractionation for the transport of COS into the cytoplasm and its degradation by COSase. The mechanisms underlying isotopic fractionation in bacterial COS degradation are also discussed using isotopic fractionation constants in addition to those of chemoorganotrophic bacteria reported previously (29).

## Materials and Methods

### Purification of COSase

COSase used in the present study was prepared as described previously (55), except that glutathione *S*-transferase (GST)-fused COSase bound to Glutathione Sepharose 4B (GE Healthcare, Chicago, IL, USA) was rinsed thoroughly, and the enzyme solution was filtered with a membrane filter before being applied to an ion exchange column. Briefly, the GST-fused COSase expression vector pCQQ15, a COSase gene-inserted pGEX-6P-1 (GE Healthcare), was transformed into *Escherichia coli* Rosetta-gami B (Merck Millipore, Billerica, MA, USA). The expression of GST-fused COSase was induced by the addition of isopropyl β-d-1-thiogalactopyranoside to the liquid culture, and cells were lysed by lysozyme. GST-fused COSase was captured by Glutathione Sepharose 4B, precipitated at 500×*g* at 4°C, and rinsed thoroughly with Factor Xa Cleavage/Capture Buffer (50 mM Tris-HCl, 100 mM NaCl, 5 mM CaCl_2_, pH 8.0) (Merck Millipore). After the digestion by Factor Xa (Merck Millipore) and capture of Factor Xa by Xarrest Agarose (Merck Millipore), the enzyme solution was filtered using a membrane filter with a pore size of 0.45 μm (Millex-HV Durapore PVDF membrane; Merck Millipore), then applied to a HiTrap Q HP column (1 mL, GE Healthcare). The purified enzyme solution was desalted and concentrated with 50 mM Tris-HCl, pH 8.5 using an Amicon Ultra-15 Centrifugal Filter Unit (Merck Millipore). Protein concentrations were estimated using Lowry’s method with bovine serum albumin as the standard (46).

### COS-degrading experiment by COSase

A 68-mL vial (V-50; Nichiden-Rika Glass, Kobe, Japan) was sealed with a butyl rubber stopper, the headspace was replaced with nitrogen, and 3.43 mL of COS (104,000 ppmv with N_2_ as the balance gas; Taiyo Nippon Sanso, Tokyo, Japan) was then added via a gastight microsyringe to make approximately 5,000 ppmv COS. After stabilizing the inside pressure using a needle, 2 mL of the enzyme solution containing 50 μg of COSase in 50 mM Tris-HCl, pH 8.5 was added to the vial and incubated at 30°C in triplicate. A control without COSase was prepared in order to monitor the non-enzymatic degradation of COS in duplicate. Sampling of the headspace gas was started 5 min after the addition of COSase in order to avoid the disturbance of the headspace gas by the addition of the enzyme solution. Sampling of the headspace gas was performed with a gastight microsyringe, and 30 μL or 50 μL and 1 mL, 2 mL, or 3 mL of the gas was used for the estimation of COS concentrations and GC-IRMS measurements, respectively. The sample for GC-IRMS was transferred into a 5-mL serum bottle (V-5B; Nichiden-Rika Glass) filled with ultrahigh purity He (>99.99995%; Taiyo Nippon Sanso).

### Bacterial culture

Fully grown *T. thioparus* THI115 (NBRC 105750) was prepared in a flask containing 10 mL of mTC10 liquid medium, mineral salts medium supplemented with thiocyanate as its sole energy source, with reciprocal shaking at 120 rpm at 30°C (34, 55). A loopful of precultured cells was inoculated on 10 mL of mTC10 agar slant medium in a glass test tube (20 cm in length, 2 cm in inner diameter). It is important to discriminate COS degradation by *T. thioparus* THI115 from the abiotic hydrolysis of COS with water (10, 12). Therefore, slant, not liquid medium was employed for the cultivation of the bacterium in order to reduce abiotic effects on the added COS as described below. After an incubation at 30°C for 25 d to achieve full growth, the COS-degrading experiment was performed.

### COS-degrading experiment by *T. thioparus* THI115

After the full growth of *T. thioparus* THI115, the silicon sponge cap was changed to a butyl rubber stopper to seal the tube in triplicate. Based on the chemolithoautotrophic characteristics of this bacterium, the headspace of 40 mL of the tube was replaced with 80% nitrogen, 20% oxygen, and 0.03% carbon dioxide. An uninoculated control was prepared to compare COS degradation in duplicate. Tubes were incubated at 30°C and the COS-degrading reaction was initiated by injecting 104,000 ppmv COS into the headspace via a gas-tight microsyringe to make approximately 4,000 ppmv COS. Sampling of the headspace gas using a gas-tight microsyringe was started 20 min after the addition of COS in order to prevent the disturbance of the headspace gas by the addition of COS. After approximately 70% of the initially added COS was degraded, the number of cells that grew on slant medium was counted. The rate constant *k* (h^−1^) of COS degradation was defined based on fitting the degradation curve to the exponential function *C*_(_*_t_*_)_=*C*_0_e^−^*^kt^*, where *C*_(_*_t_*_)_ is the concentration of COS at time *t* (h) and *C*_0_ is the initial COS concentration.

### COS concentration measurements

COS concentrations were estimated by directly injecting the headspace gas into a gas chromatograph (GC-14B; Shimadzu, Kyoto, Japan) as described previously (29). Briefly, the gas chromatograph was equipped with a flame photometric detector and glass column (2.0 mm in length, 3.0 mm in inner diameter) packed with Sunpak-S (Shimadzu). N_2_ gas (Ichimura Sanso, Tokyo, Japan) was used as the carrier gas at a flow rate of 100 mL min^−1^. The temperatures for the injector, column, and detector were 190°C, 60°C, and 190°C, respectively. COS concentrations were corrected by taking into account decreases caused by sampling. The relative standard deviation was within 3%.

### Stable isotope measurement

The sulfur isotopic composition of COS was measured using online GC-IRMS with measurements of the fragment ion S^+^ from COS, as developed previously (22). Briefly, the carrier gas was He (>99.99995% purity; Taiyo Nippon Sanso) treated with a 5 A Molecular Sieve (Sigma-Aldrich, Tokyo, Japan) to remove any trace contaminants. An aliquot (an amount larger than 8 nmol) of COS in a 5-mL serum bottle was initially trapped in a stainless-steel tube chilled with liquid N_2_. COS was then desorbed by bringing back to room temperature and transferred by a He flow to a capillary tube chilled with liquid N_2_. Preconcentrated COS was desorbed by removing the tube from liquid N_2_ and introduced into the GC equipped with a capillary column (30 m in length, 0.32 mm in inner diameter, and with a thickness of 10 μm, HP-PLOT Q; Agilent Technologies, Santa Clara, CA, USA), and then IRMS (MAT 253; Thermo Fisher Scientific, Bremen, Germany) was performed to measure the fragment ions ^32^S^+^, ^33^S^+^, and ^34^S^+^. Approximately 11 ppmv of COS (Japan Fine Products, Kawasaki, Japan) was used to confirm the accuracy and precision of measurements and the standard deviations for *δ*^33^S, *δ*^34^S, and Δ^33^S values of the triplicate measurements were within 0.1‰, 0.1‰, and 0.1‰ during the experimental period of COSase, and 0.6‰, 0.3‰, and 0.5‰ during the experimental period of *T. thioparus* THI115, respectively.

### Cell number

Living and dead cells were enumerated microscopically after live/dead staining (LIVE/DEAD BacLight Bacterial Viability Kit for microscopy; Thermo Fisher Scientific), as described previously (29). Briefly, cells on slant medium were suspended in 0.85% NaCl, collected by centrifugation at 10,000×*g* for 15 min, and then rinsed twice as described. The pellet was resuspended in 0.85% NaCl and Live/Dead reagent was added to 1 mL of the diluted cell suspension. After 15 min, the cell suspension was filtered onto a polycarbonate black filter (0.2 μm pore size and 25 mm in diameter; Advantec Toyo Kaisha, Tokyo, Japan) treated with 0.01% poly-l-lysine (P8920; Sigma-Aldrich). The cell number was enumerated under a fluorescence microscope (BZ-8000; KEYENCE, Osaka, Japan).

### Definition

The isotopic compositions of ^33^S and ^34^S values were expressed in δ notations as

δSx=Rsample/Rreference-1

where *R*_sample_ and *R*_reference_ are the isotope ratios (*^x^*S/^32^S, where *x*=33 or 34) of residual COS at the times indicated and initial COS, respectively. In addition, Δ^33^S value was used to distinguish between mass-dependent fractionation (MDF) and mass-independent fractionation (MIF; or non-mass-dependent fractionation) and defined as deviations from the MDF line, as expressed in the following equation:

ΔS33=δS33-[(δS34+1)0.515-1]

The magnitude of isotopic fractionation during a single unidirectional reaction was expressed using the isotopic fractionation factor *α*,

α=kx/k32

where *^x^k* (*x*=33 or 34) and ^32^*k* are the reaction rates for the heavy and light isotopes, respectively. The isotopic fractionation constant *^x^ɛ* (*x*=33 or 34) is defined as follows:

ɛx=(αx-1)

On the other hand, *^x^ɛ* can be estimated from the Rayleigh equation according to Mariotti *et al.* (47),

δSx-δSxinitial=ɛxlnf

where *f* is defined as the ratio of the residual COS concentration at the times indicated divided by the initial COS concentration. MIF can be described as a deviation from MDF in ^33^S (^33^*E*) defined by the MDF law (1, 24), as follows:

E33=ɛ33-0.515ɛ34

### Statistical analysis

The rate constant *k* of COS degradation was calculated using the function SLOPE from Excel for Mac 2011 (version 14.7.2, Microsoft, Redmond, WA, USA). Free software R (version 3.4.1) (R Core Team. 2017. R: A Language and Environment for Statistical Computing. R Foundation for Statistical Computing, Vienna, Austria. URL https://www.R-project.org/) was used to calculate and analyze statistics for the *ɛ* and ^33^*E* values for COS degradation by COSase and *T. thioparus* THI115. The slope between *δ*^33^S or *δ*^34^S values and ln*f* shows the *ɛ* value, and the *ɛ* value±SD was calculated based on the least square method passing through an origin using the function summary from R. The resultant values were shown as the mean±SD in triplicate. A *t*-test was performed for the comparison of *ɛ* values between COSase and *T. thioparus* THI115 using the function t.test from R. *P* values<0.05 were considered to indicate significance.

## Results and Discussion

### Sulfur isotopic fractionation for COS degradation by COSase

**Fig. 1:**
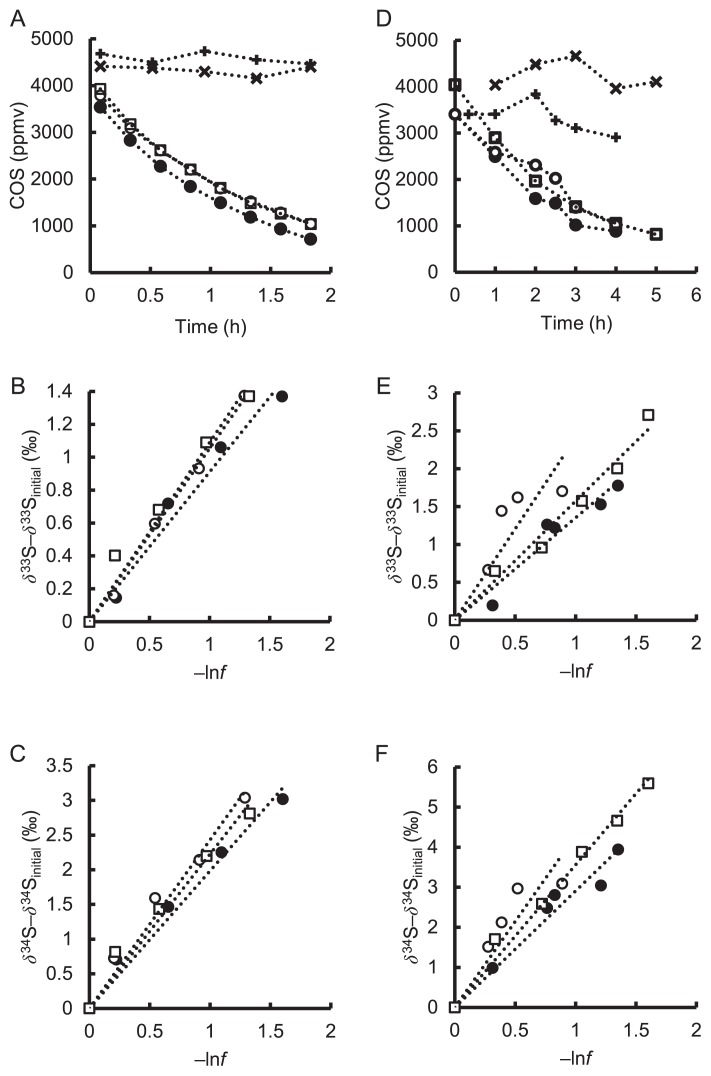
Time courses of COS and sulfur isotopic compositions during COS degradation by COSase (A, B and C) and *T. thioparus* THI115 (D, E and F). ln*f* represents the natural logarithm of the ratio of the residual COS concentration at the times indicated divided by the initial COS concentration. (A and D) ●, ○, and □ represent batches 1, 2, and 3 of COSase or *T. thioparus* THI115, respectively. × and + represent batches 1 and 2 of buffer without COSase or the uninoculated control, respectively. (B, C, E and F) ●, ○, and □ represent batches 1, 2, and 3 of *T. thioparus* THI115 or COSase, respectively. The COS concentration at 0 min cannot be measured because of the disturbance of the headspace gas by the addition of COS. Therefore, the concentration of *T. thioparus* THI115 at 0 min was regarded as those of batches 1 and 2 of the uninoculated control measured at 20 min and 60 min, respectively, corresponding to the date of the measurement.

COSase degraded more than 70% of the initially supplemented COS within 2 h under the experimental conditions employed (Fig. 1A). Degradation curves fit the exponential function and the rate constant was 0.79±0.10 h^−1^ (*r*^2^=1.00), indicating that COS degradation by COSase was a first order reaction within the range of the COS concentrations used in the present study (Fig. 1A and Table 1). On the other hand, control batches without COSase showed slight variations in COS concentrations (Fig. 1A). Since no H_2_S was detected, we attributed these variations to experimental errors, not chemical hydrolysis. COS degradation by COSase relative to the control batches was very fast; therefore, variations were not considered to affect the assessment of isotopic fractionation. The *δ*^33^S and *δ*^34^S values in residual COS increased during COS degradation, and the slope between *δ*^33^S or *δ*^34^S values and ln*f* showed that ^33^*ɛ* and ^34^*ɛ* values were −1.0±0.1‰ and −2.2±0.2‰, respectively (Fig. 1B and C, and Table 1).

The purified enzymes studied to date in sulfur isotopic fractionation have only been DsrAB and *s*-triazine hydrolase (TrzN) (43, 67), and reported ^34^*ɛ* values were −15.3‰ and −16‰ for sulfite reduction by DsrAB from *Desulfovibrio vulgaris* and *Archaeoglobus fulgidus*, respectively. The ^34^*ɛ* value for TrzN from *Arthrobacter aurescens* was −14.7‰ for the substitution of the thiomethyl group in the herbicide ametryn to a hydroxyl group. In contrast to these enzymes, COSase showed smaller isotopic fractionation. The active site of TrzN had a similar X-ray crystal structure to that of α-CA; however, their amino acid sequences and overall folding structures were different (69). The conformations of the active site between β-CA including COSase and α-CA are similar to each other; however, the COS-degrading activity of α-CA is lower than that of β-CA (20, 39, 57). Although the substrates for COSase and TrzN differ, a common reaction mechanism has been proposed for these enzyme proteins, as follows: a nucleophilic attack to the carbon atom by the hydroxide ion coordinated with the zinc ion at the active site, followed by cleavage of the C-S bond in the substrate (55, 67). Schürner *et al.* (67) proposed that C-S bond cleavage, which was rate-limiting for the energetic barrier, was the reason for the large sulfur isotope effect by TrzN. On the other hand, a computational model in the reaction of COS with ([H_3_N]_3_ZnOH)^+^, a biomimetic complex used to mimic the active site of α-CA, showed that the rate-limiting step was the nucleophilic attack (66). Therefore, we hypothesize that the factor controlling isotopic fractionation by COSase may be the rate-limiting reaction by the nucleophilic attack to the carbon atom of COS. In other words, since cleavage of the C-S bond in COS was not the rate-limiting step in the hydrolysis of COS, cleavage mostly proceeded and did not show isotopic fractionation for the cleavage reaction itself.

### COS degradation by *T. thioparus* THI115

*T. thioparus* THI115 degraded approximately 70% of initial COS in 4 h (Fig. 1D). Cell numbers on slant medium in culturing tubes supplemented with COS were in the range of 4.7×10^9^ to 2.0×10^9^ cells tube^−1^ for living cells and 4.8×10^9^ to 1.3×10^9^ cells tube^−1^ for dead cells. Cell numbers in the COS free slant tube were in the range of 3.1×10^9^ to 3.2×10^9^ cells tube^−1^ for living cells and 8.1×10^8^ to 1.8×10^9^ cells tube^−1^ for dead cells, suggesting that cells were not negatively affected by the COS treatment. Degradation curves were fit to the exponential function and the rate constants were 0.33±0.03 h^−1^ (*r*^2^≥0.94), showing that COS degradation is a first order reaction (Fig. 1D and Table 1). The cell-specific activities (rate constant divided by cell number) of *T. thioparus* THI115 were in the range of 0.76 to 1.51×10^−10^ h^−1^ cell^−1^ (Table 1). Among bacteria examined to date, *T. thioparus* THI115 has exhibited the highest COS-hydrolyzing activity; the specific activities of bacterial isolates already examined have been in the range of 0.07 to 1.12×10^−10^ h^−1^ cell^−1^ (29). On the other hand, uninoculated control batches showed variations in COS concentrations, as did control batches for COS degradation by COSase. However, H_2_S was not observed in this case, and the reason for the variations observed beyond the measurement error currently remains unclear. The isotopic fractionation of an abiotic decrease in COS was not assessed because COS degradation by *T. thioparus* THI115 was markedly faster than that by the control batches.

*T. thioparus* THI115 is a sulfur-oxidizing bacterium that degrades COS and H_2_S as the chemolithotrophic energy source. This bacterium also tolerates high concentrations of COS because the addition of 1,500 ppmv and 25,000 ppmv of COS did not negatively affect the degradation of thiocyanate or COS, respectively (38). The high activity observed by *T. thioparus* THI115 is appropriate because the COS-degrading reaction is associated with the energy production pathway for this bacterium. On the other hand, the physiological functions of the COS-degrading activity of various chemoorganotrophic bacteria (36, 55) and fungi (44, 48) have not yet been elucidated in detail. Approximately half of these bacteria contain a gene of clade D of β-CA, one of four clades into which β-CA is phylogenetically classified (73, 74). COS degradation may only accompany the reversible hydration of CO_2_, the natural substrate of β-CA, to HCO_3_^−^ and H^+^ by β-CA because COS is a structural analog of CO_2_.

### Sulfur isotopic fractionation for *T. thioparus* THI115

*δ*^33^S and *δ*^34^S values for residual COS increased during COS degradation by *T. thioparus* THI115, as was also the case for COSase (Fig. 1E and F). The ^33^*ɛ* and ^34^*ɛ* values estimated from the slope were −1.8±0.6‰ and −3.6±0.7‰, respectively (Fig. 1E and F, and Table 1), while ^33^*E* values were 0.1±0.1‰ for COSase and 0.1±0.2‰ for *T. thioparus* THI115 (Table 1). We herein demonstrated for the first time that ^34^*ɛ* values for *T. thioparus* THI115 and COSase were different (*P* value<0.05). Most errors in isotope ratio measurements were caused by MDF, and variations in ^33^*E* values were less than those for ^33^*ɛ* and ^34^*ɛ* values (Table 1). Since the interpretation of ^33^*E* values will be discussed in the next section, we only focused on the ^34^*ɛ* values for *T. thioparus* THI115 and COSase in this section.

**Fig. 2:**
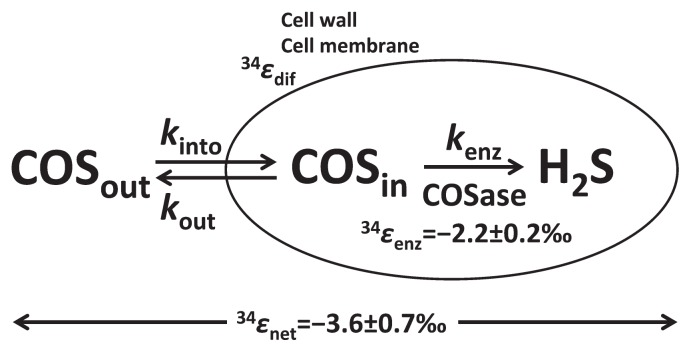
Schematic diagram of ^34^S isotopic fractionation by *T. thioparus* THI115. *ɛ*_net_, *ɛ*_dif_, and *ɛ*_enz_ represent the isotopic fractionation constants for the overall net, transport into the cytoplasm, and the enzyme reaction, respectively. *k*_into_, *k*_out_, and *k*_enz_ represent rate constants for diffusion into and out of the cell of COS and degradation by enzymes, respectively.

In order to elucidate the mechanisms and factors controlling sulfur isotopic fractionation for COS degradation, we considered Rees’ model (62). The cell membrane is one of the important factors for isotopic fractionation by diffusion and/or active transport (21, 58). Based on Rees’ model, isotopic fractionation in COS degradation by intact *T. thioparus* THI115 reflects effects on COS transport into the cytoplasm, the hydrolysis of COS by COSase, and the ratio of efflux to influx [*k*_out_/(*k*_out_+*k*_enz_) or *k*_out_/*k*_into_] (Fig. 2). This relationship can be expressed using Rees’ model (62), as follows:

$${ɛ}_{\text{net}}={ɛ}_{\text{dif}}+({ɛ}_{\text{enz}}-{ɛ}_{\text{dif}})k_{\text{out}}/(k_{\text{out}}+k_{\text{enz}})$$

where *ɛ*_net_, *ɛ*_dif_, and *ɛ*_enz_ are isotopic fractionation constants for the overall net, transport into the cytoplasm, and the enzyme reaction, respectively. *k*_into_, *k*_out_, and *k*_enz_ are rate constants for transport into and out of the cell and for COS degradation by an enzyme, respectively. Transport is regarded as diffusion into and out of the cytoplasm through the cell wall and cell membrane because COS is a gaseous sulfur compound and the cytoplasm is in the liquid phase. Diffusivity in the liquid phase of one gas in another is noted as *D*_T_, and is calculated as follows (52):

$$D_{\text{T}}={\left(\frac{\left(m_{1}\;m_{2}\right)}{\left(m_{1}+m_{2}\right)}\right)}^{-\frac{1}{2}}.$$

In this equation, *m*_1_ and *m*_2_ represent the masses of a gas and liquid, respectively. Thus, *D*(CO^32^S) and *D*(CO^34^S) can be calculated based on the masses of CO^32^S, CO^34^S, and H_2_O. The ^34^*ɛ*_dif_ value for COS diffusion in liquid water is calculated as *D*(CO^34^S)/*D*(CO^32^S)−1, and the value is −3.7‰. Thus, the model shows the upper limit of ^34^*ɛ*_net_=−3.7‰ when the ratio of efflux to influx is equivalent to 0 (*i.e. k*_out_<<*k*_enz_), while the lower limit of ^34^*ɛ*_net_=−2.2‰, ^34^*ɛ* value of COSase, when the ratio of efflux to influx is equal to 1 (*i.e. k*_out_>>*k*_enz_). Thus, the ^34^*ɛ*_net_ value of −3.6±0.7‰ obtained in the present experiment using *T. thioparus* THI115 suggests that the ratio of efflux to influx of this experiment was 0.07, *k*_into_ was 0.35 h^−1^, and *k*_enz_ (0.33 h^−1^) was much higher than *k*_out_ (0.02 h^−1^). In other words, the main factor controlling the ^34^*ɛ* value of bacterial COS degradation was not the enzyme, but diffusion/transport into the cytoplasm. We are the first to show *k*_into_ and *k*_out_ needed in order to estimate influx and efflux for COS degradation by *T. thioparus* THI115.

In the present study, we employed a higher concentration of COS than the atmosphere because the IRMS analysis required more than 8 nmol COS for technical reasons (22). Based on the emission rate of 13 pmol m^−2^ s^−1^ COS from a wheat farm after harvesting (49, 78), Sun *et al.* (78) estimated that the COS concentration in the soil was approximately 10 parts per billion by volume (ppbv). The highest COS flux in the soil environment reported to date was ~50 pmol m^−2^ s^−1^ and was observed at a wheat farm (2); therefore, COS concentrations are presumed to be within an order of ppbv. Isotopic fractionation for *T. thioparus* THI115 was defined by *ɛ* values for the transport of COS into the cytoplasm, the enzyme reaction by COSase, and the ratio of efflux to influx. We assumed that the ^34^*ɛ*_net_ value for *T. thioparus* THI115 under very low COS concentrations, such as the atmosphere, was close to the value obtained under high COS concentrations because the ratio of efflux to influx is almost 0, and also that ^34^*ɛ*_net_ value was very close to −3.7‰ based on Rees’ model (62).

We previously reported ^34^*ɛ* values for seven strains of bacteria isolated from soil. ^34^*ɛ* values for four isolates of *Mycobacterium* spp. and *Williamsia* sp. THI410, both of which belong to the phylum *Actinobacteria*, were −3.99‰ to −3.56‰ and −3.74‰, respectively. ^34^*ɛ* values for *Cupriavidus* spp. THI414 and THI415, bacteria belonging to the phylum *Proteobacteria*, were −2.09‰ and −2.38‰, respectively (29). The reason why the ^34^*ɛ* value for *T. thioparus* THI115 was closer to those for the five isolates of *Actinobacteria* may be explained by similarities in the function of COS transport into the cytoplasm, enzymes, and the resultant ratio of efflux to influx, which are factors affecting isotopic fractionation based on Rees’ model (62).

It is important to note that different enzymes for COS degradation may also partially explain the difference in ^34^*ɛ* values among different species. COSase examined here belongs to clade D of β-CA (55). Genome information on *Mycobacterium* spp. THI401, THI402, THI404, and THI405 and *Williamsia* sp. THI410 suggests that these bacteria have clade D of β-CA because these strains have similar partial nucleotide sequences to that of the gene encoding clade D of β-CA (56). In contrast, these sequences were not detected in the genome of *Cupriavidus* spp. THI414 or THI415 (56, unpublished data for *Cupriavidus* sp. THI414). In addition, NCBI’s CD-Search, a search tool for conserved domains, indicated that only 2 out of 245 CAs of *Cupriavidus* spp. searched from the NCBI’s protein database were classified to clade D of β-CA, suggesting that *Cupriavidus* spp. THI414 and THI415 do not have β-CA classified in clade D. Although the number of bacterial isolates examined here was limited, our results suggest that the ^34^*ɛ* value for bacteria is closely related to the presence of relevant enzymes.

COS-degrading ability is widespread not only in bacteria (36, 56) and archaea (71), but also in plants (61), fungi (44, 48), and algae (3, 60). Due to high COS-degrading activities (55, 57, 71, 72) and the distribution of β-CA family enzymes, which include COSase and CS_2_ hydrolase, in phylogenetically diverse organisms (73, 74), COS degradation by these organisms appears to mainly be mediated by β-CA family enzymes. Therefore, isotopic fractionation for COS-degrading organisms is considered to be similar to COSase at the enzyme level, indicating that the lower limit of the ^34^*ɛ* value for these organisms is also similar to that for COSase.

On the other hand, the presence of other COS-degrading enzymes needs to be considered. It is unlikely that *T. thioparus* THI115 possesses other β-CA family enzymes because a Southern blotting analysis using the COSase gene showed the presence of only one copy of the COSase gene in the genome (55). However, we are still unable to rule out the possible effects of other COS-degrading enzymes such as α-CA (20), nitrogenase (68), CO dehydrogenase (11), and RubisCO (45), which exhibit low COS-degrading activities. The recently elucidated draft genome of *T. thioparus* DSM 505 indicated that there are nucleotide sequences annotated to β-CA, carboxysome CA, γ-CA, and RubisCO (25). The X-ray crystal structures of the active sites of carboxysome CA and γ-CA were similar to those of β-CA and α-CA, respectively (23, 40, 65). The genome sequencing of *T. thioparus* THI115 is in progress in order to obtain information on the involvement of these enzymes.

### Three-isotope plot for COS degradation by COSase and *T. thioparus* THI115

**Fig. 3:**
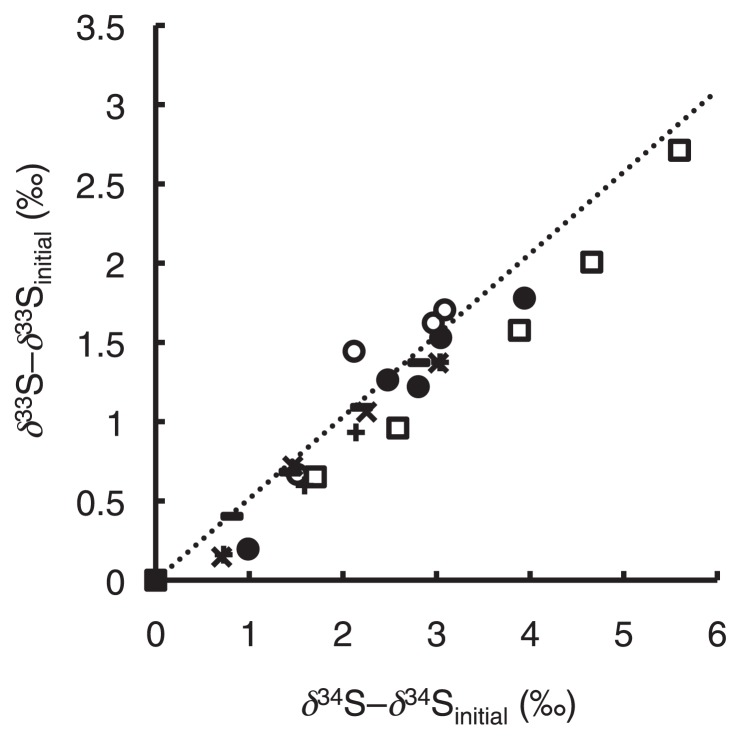
A three-isotope plot between *δ*^33^S and *δ*^34^S values. The broken line indicates the mass-dependent fractionation line with slope (0.515). ×, +, and − represent batches 1, 2, and 3 of COSase, respectively. ●, ○, and □ represent batches 1, 2, and 3 of *T. thioparus* THI115, respectively.

Fig. 3 shows a three-isotope plot represented from the results of the present study, and slopes ranged between 0.43 and 0.56. Since most errors in isotope ratio measurements were caused by MDF, variations in ^33^*E* values were less than those for ^33^*ɛ* and ^34^*ɛ* values (Table 1). ^33^*E* values were 0.1±0.1‰ for COSase and 0.1±0.2‰ for *T. thioparus* THI115 (Table 1), showing no significant difference.

Sulfur isotope anomalies originating from the MIF process have been observed in aerosols from the troposphere and in snow pits and ice cores in which aerosols from the stratosphere have been deposited (17, 63, 64). The reactions by COSase and *T. thioparus* THI115 as well as chemoorganotrophic bacteria reported previously did not show MIF, which confirmed that these reactions do not contribute to sulfur isotope anomalies in the atmosphere (17, 63, 64).

## Conclusion

Isotopic fractionation for COSase and *T. thioparus* THI115 in the present study indicated biological effects on *δ*^34^S value of tropospheric COS and provides insights into biological COS degradation, which is important for estimating the COS global budget. In the present study, we used a purified enzyme of COSase and intact cells of *T. thioparus* THI115 in order to obtain a clearer understanding of isotopic fractionation in COS degradation by bacteria. Based on Rees’ model, we assumed that isotopic fractionation by *T. thioparus* THI115 was mainly influenced by the transport of COS into the cytoplasm during the degradation of COS, even at very low concentrations such as atmospheric COS.

Further studies with a focus on isotopic fractionation by plants, another major sink of atmospheric COS, are indispensable for distinguishing COS fluxes between soil and plants. Moreover, measurements at low COS concentrations, such as atmospheric concentrations, are needed for soil, the second largest sink of COS, and COS-degrading organisms in order to clarify the COS dynamics operating between the troposphere and soil or organisms (42). Therefore, a rapid and simple analytical method with the ability to measure isotopic fractionation at atmospheric COS levels needs to be developed.

## Figures and Tables

**Table 1:** Sulfur isotopic fractionations in COS degradation by COSase and *T. thioparus* THI115.^a^

	Batch	Isotope sampling times	Rate constant (h^−1^)	Living cell number (×10^9^ cell)	Cell specific activity (×10^−10^ h^−1^ cell^−1^)	^33^*ɛ*			^34^*ɛ*			
		
						(*‰*)	*r*^2^	*P* value	(*‰*)	*r*^2^	*P* value	^33^*E* (*‰*)
COSase	1	5	0.90	—	—	−0.9±0.1	0.99	2.8×10^−5^	−2.0±0.2	0.99	2.2×10^−5^	0.1
	2	5	0.72	—	—	−1.1±0.0	1.00	7.6×10^−7^	−2.4±0.2	0.99	2.6×10^−5^	0.2
	3	5	0.75	—	—	−1.1±0.1	0.99	4.1×10^−5^	−2.2±0.3	0.99	4.0×10^−5^	0.1
	Average^b^	—	0.79±0.10	—	—	−1.0±0.1	—	—c	−2.2±0.2	—	—c	0.1±0.1

*T. thioparus*	1	6	0.36	4.7	0.76	−1.4±0.2	0.99	9.1×10^−6^	−2.9±0.3	0.99	4.8×10^−6^	0.1
THI115	2	5	0.30	2.0	1.51	−2.4±0.8	0.93	2.0×10^−3^	−4.3±1.3	0.94	1.2×10^−3^	−0.2
	3	6	0.33	ND	ND	−1.6±0.1	0.99	1.3×10^−6^	−3.6±0.3	1.00	3.9×10^−7^	0.3
	Average^b^	—	0.33±0.03	3.3±1.9	1.14±0.53	−1.8±0.6	—	—c	−3.6±0.7	—	—c	0.1±0.2

ND, not determined.

a Indicated ±SD.

b Indicated mean±SD calculated based on the value of each batch.

c *P* values for *ɛ* values between COSase and *T. thioparus* THI115 were 0.08 for the ^33^*ɛ* value and 0.03 for the ^34^*ɛ* value.
